# Severe Fever With Thrombocytopenia Syndrome Virus-Induced Macrophage Differentiation Is Regulated by miR-146

**DOI:** 10.3389/fimmu.2019.01095

**Published:** 2019-05-15

**Authors:** Li Zhang, Yuxuan Fu, Huanru Wang, Yajie Guan, Weiwen Zhu, Mengdi Guo, Nan Zheng, Zhiwei Wu

**Affiliations:** ^1^Center for Public Health Research, Medical School, Nanjing University, Nanjing, China; ^2^Jiangsu Key Laboratory of Oral Diseases, Nanjing Medical University, Nanjing, China; ^3^State Key Lab of Analytical Chemistry for Life Science, Nanjing University, Nanjing, China; ^4^Jiangsu Key Laboratory of Molecular Medicine, Medical School, Nanjing University, Nanjing, China

**Keywords:** SFTSV, macrophage differentiation, miR-146, interleukin 10, STAT1

## Abstract

Severe fever with thrombocytopenia syndrome (SFTS) is an emerging hemorrhagic fever with a high mortality rate in humans, which is caused by SFTS virus (SFTSV), a novel phlebovirus in the *Bunyaviridae* family, is tick borne and endemic in Eastern Asia. Previous study found that SFTSV can infect and replicate in macrophages *in vivo* and *in vitro*. However, the role of macrophages in virus replication and the potential pathogenic mechanisms of SFTSV in macrophage remain unclear. In this study, we provided evidence that the SFTSV infection drove macrophage differentiation skewed to M2 phenotype, facilitated virus shedding, and resulted in viral spread. We showed evidence that miR-146a and b were significantly upregulated in macrophages during the SFTSV infection, driving the differentiation of macrophages into M2 cells by targeting STAT1. Further analysis revealed that the elevated miR-146b but not miR-146a was responsible for IL-10 stimulation. We also found that SFTSV increased endogenous miR-146b-induced differentiation of macrophages into M2 cells mediated by viral non-structural protein (NSs). The M2 skewed differentiation of macrophages may have important implication to the pathogenesis of SFTS.

## Introduction

Severe fever with thrombocytopenia syndrome (SFTS) is characterized by hemorrhagic fever with a high mortality rate in humans, which is caused by SFTS virus (SFTSV), a novel phlebovirus in the *Bunyaviridae* family. The most common clinical symptoms of SFTS include fever, nausea, diarrhea, myalgia, and bleeding with 12–30% of clinical mortality (1, 2). People working in rural areas, especially farmers, hospital staff attending to positive SFTSV patients, and those with high levels of interaction with animals are at risk of acquiring SFTSV infection (3, 4). Monocytes/macrophages are believed to be the main targets of SFTSV infection (5, 6). In addition to their roles in clearance of circulating virus by phagocytosis (6, 7), macrophages play critical roles in innate immune defense by modulating adaptive immune response to various pathogens through antigen processing and presentation (8–10). Upon infection, macrophages differentiate into two functional subsets M1 and M2 with distinct phenotypes. M1-macrophages are characterized as pro-inflammatory and tissue destructive. In contrast, M2-macrophages are anti-inflammatory and tolerogenic (11–13) and are characterized by increased phagocytic activity but suppressed production of proinflammatory cytokines and reduced killing capacity toward pathogens (14). Studies have shown that macrophages are stimulated to skew toward M2 phenotype by viral infection (15, 16). Indeed, most monocyte tropic viral infections, such as those caused by HIV, RSV, SARS, and IAV, may affect macrophage polarization, and in turn oblige the host with the outcome of immunosuppression and/or immunopathology; these processes are generally associated with viral persistence and co-infections by pathogens of other phyla (17). Depending on the activating stimulus received, M2 macrophages can be further divided into four different subsets consisting of M2a, M2b, M2c, and M2d (18). The M2a subset of macrophages could be induced by IL-4 and IL-13 and produces high levels of CD206, decoy receptor IL-1 receptor II (IL-RII), and IL-1 receptor antagonist (IL1Ra) (19). The M2b subset could be induced by stimulation with immune complexes (ICs) and Toll-like receptor (TLR) agonists or IL-1 receptor ligands (19). M2b macrophages produce both anti- and proinflammatory cytokines IL-10, IL-1β, IL-6, and TNF-α (18). M2c subset is induced by glucocorticoids and IL-10 and exhibits strong anti-inflammatory activities against apoptotic cells by releasing high levels of IL-10 and TGF-β (18, 20). Finally, a fourth type of M2 macrophage, M2d, is induced by TLR agonists through the adenosine receptor (19). The classical pathway of IFN-γ-dependent activation of macrophages by T helper 1 (T(H)1)-type responses is a well-established feature of cellular immunity to intracellular pathogens, such as mycobacterium tuberculosis and HIV (14). The concept of an alternative pathway of macrophage activation by the T(H)2-type cytokines IL-4 and IL-13 has gained credence in the past decade, to account for a distinctive macrophage phenotype that is consistent with a different role in humoral immunity and repair (14).

Macrophages can present antigens to and activate T lymphocytes. Two important co-stimulatory molecules are the cell-surface proteins B7.1 (CD80) and B7.2 (CD86), which are induced on macrophages and tissue dendritic cells by innate sensors in response to pathogen recognition. B7.1 and B7.2 are recognized by specific co-stimulatory receptors expressed by cells of the adaptive immune response, particularly CD4 T cells, and their activation by B7 is an important step in adaptive immune responses. CD4 T-cell depletion in SFTS patients and increased Th2 and Th17-cell percentages in the residual CD4 T-cell population led to aberrant Th2/Th1 and Th17/Treg ratios, which were positively correlated with disease severity.

Accumulating evidences have shown that microRNAs (miRNA), a conserved class of endogenous non-coding RNAs that modulate the post-transcriptional expression of specific genes, can regulate macrophage polarization and subsequent effects on inflammation (21, 22). Several miRNAs have been shown to be associated with polarized macrophages. Usually, they regulate the expression of various adaptor proteins and transcription factors, which are known to participate in macrophage polarization (23, 24). Thus, the alteration of such miRNA levels in macrophages may affect the switch between M1 and M2 phenotypes (25–27). For instance, miR-127 and miR-155 can promote M1 polarization, while miR-223, miR-34a, and miR-125a-5p, can induce M2 polarization in both circulatory monocytes and tissue-resident macrophages (28, 29). Several targets of miR-155 have been identified in macrophages, including suppressor of cytokine signaling 1 (SOCS1) and B cell leukemia/lymphoma 6 (Bcl6), which mediate the pro-inflammatory effects of miR-155 (30, 31). The anti-inflammatory M2 microRNA, miR-223-3p, limits IL-1b protein expression by targeting the inflammasome component Nlrp3 in macrophages (32). Several targets of miR-223-3p have been identified in macrophages, including the Pbx/knotted 1 homeobox (Pknox1, also known as Prep-1), RAS p21 protein activator (GTPase activating protein) 1 (RASA1), nuclear factor of activated T cells 5 (NFAT5), STAT3, and IKKa, which might mediate the anti-inflammatory effects of miR-223-3p (33, 34). However, the roles of miRNAs in macrophage differentiation during viral infection are poorly documented. In the current study, we showed that SFTSV infection significantly upregulated miR-146a/b expression in macrophages, which resulted in the increase of macrophage differentiation into M2 phenotype through miR-146a/b targeting of STAT1. Mechanistic analysis showed that IL-10, an immune suppressive cytokine that is upregulated in SFTSV infection, stimulated miR-146b but not miR-146a expression.

THP-1 cells express characteristics of monocytes and are widely used as a model cell line for monocytes (35). The enhanced clearance of virus-bound platelets promoted by splenic macrophages appears to be the major cause of thrombocytopenia in SFTSV-infected mice. SFTSV can directly infect macrophages and is harbored within splenic macrophages for long periods (6). The role of macrophages in limiting virus replication is current unknown and is being investigated in our lab for the potential pathogenic mechanisms.

## Results

### SFTSV Infection Induced Macrophage Differentiation

**Figure 1:**
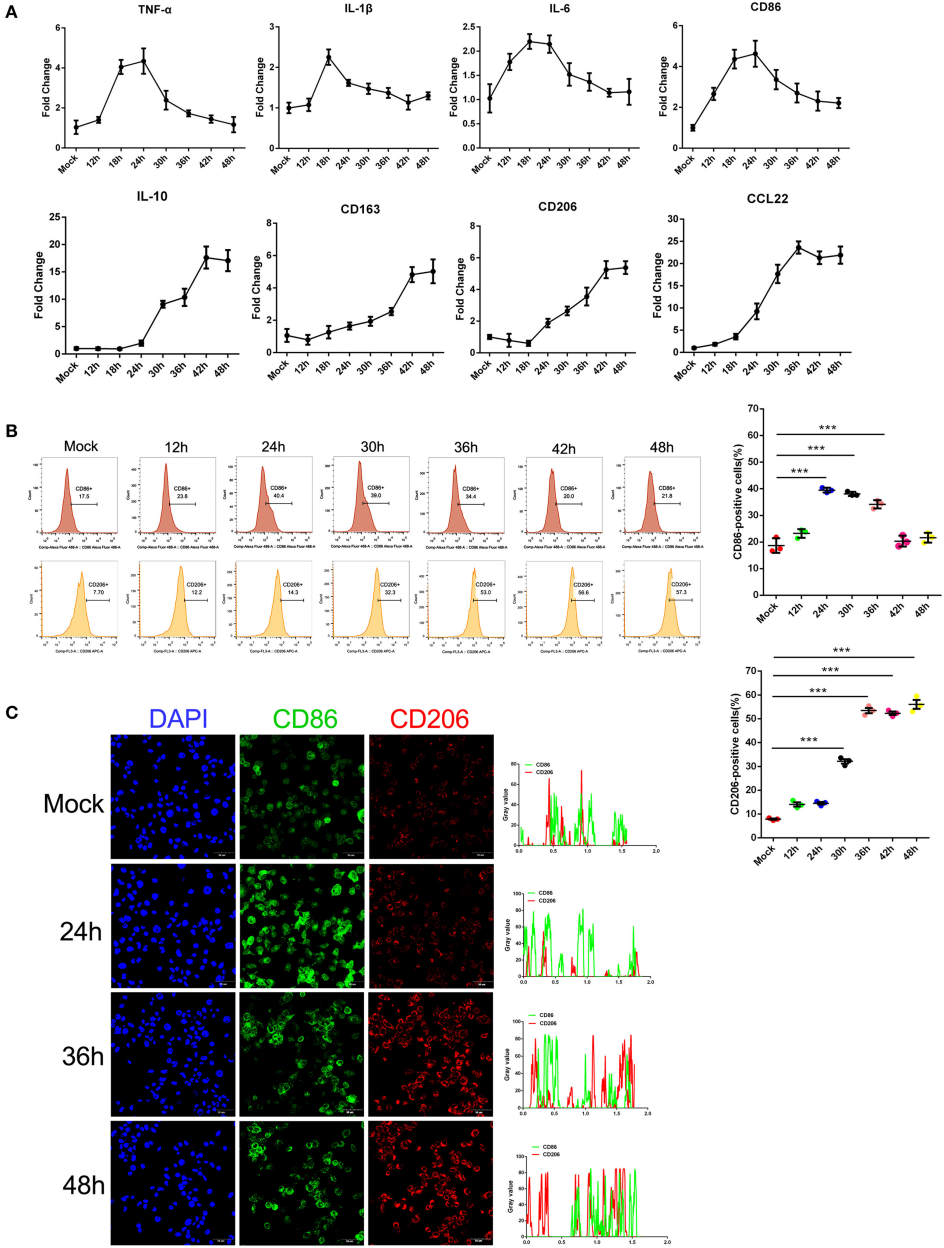
SFTSV infection induced macrophage differentiation. **(A)** Real-time PCR analysis of M1 (TNF-α, IL-1β, IL-6, and CD86), and M2 markers (IL-10, CD163, CD206, and CCL22) of THP-1 cells infected for various time durations. **(B)** Flow-cytometry profiling of M1 (CD86^+^) and M2 (CD206^+^) macrophage subsets within a gated CD11b^+^ population by FACS analysis. Percentage change of CD86^+^ cells (upper panel) and CD206^+^ cells (lower panel) is depicted. **(C)** Fluorescence analysis of M1 marker CD86 (green) and M2 marker CD206 (red) in SFTSV-infected THP-1 cells at various time points. Right graph: fluorescence intensity of CD86 (green) and CD206 (red) in the regions delineated by a white line through ImageJ software. In ImageJ software, pixel counts are used to determine the differences in fluorescence intensity. The value profiles represent fluorescence intensity of CD86 (green) and CD206 (red), which could reflect the expression levels of these two proteins. Bar = 50 μm. All data are shown as mean ± SEM of three independent experiments (^***^P < 0.001).

Earlier studies by others and our group showed that monocytes are the prime target for SFTSV infection and the differentiation of monocytes may be impeded because of the infection (6). To investigate the impact of SFTSV infection on macrophage differentiation, we infected human monocytic cell line THP-1 and analyzed the cells by real-time PCR for phenotypic markers at various time points. Macrophage M1 differentiation was assessed by measuring the expression of several classical M1 markers: TNF-α, IL-1β, IL-6, and CD86, while M2 by IL-10, CD163, CD206, and CCL22. The mRNA level of these M1 markers increased in the first 12–24 h post-infection and then decreased gradually after 24 h. In contrast, the mRNA level of M2 markers steadily increased upon viral infection and peaked at 42 h post-infection (Figure 1A). These data showed that infection by SFTS virus drives infected THP-1 differentiation toward first an pro-inflammatory M1 phenotype characterized by increase of TNF-α, IL-1β, IL-6, and CD86 mRNA within the first 12–24 h followed by change to the anti-inflammatory M2 phenotype characterized by CD163, CD206, IL-10, and CCL22 mRNA expression. In addition, the expression of macrophage markers CD86 and CD206 was confirmed at the protein level by FACS analysis. CD86 expression increased during the early stage of infection and then decreased gradually after 24 h post-infection; however, the expression of the CD206 increased from 12 hpi and continued to 48 hpi (Figure 1B), consistent with the real-time PCR results. Immunofluorescence staining of the infected cells showed the same patterns (Figure 1C). Together, these data demonstrated that SFTSV infection drives macrophage differentiation skewed to M2 phenotype.

### Macrophage Differentiation Affected SFTSV Replication

**Figure 2:**
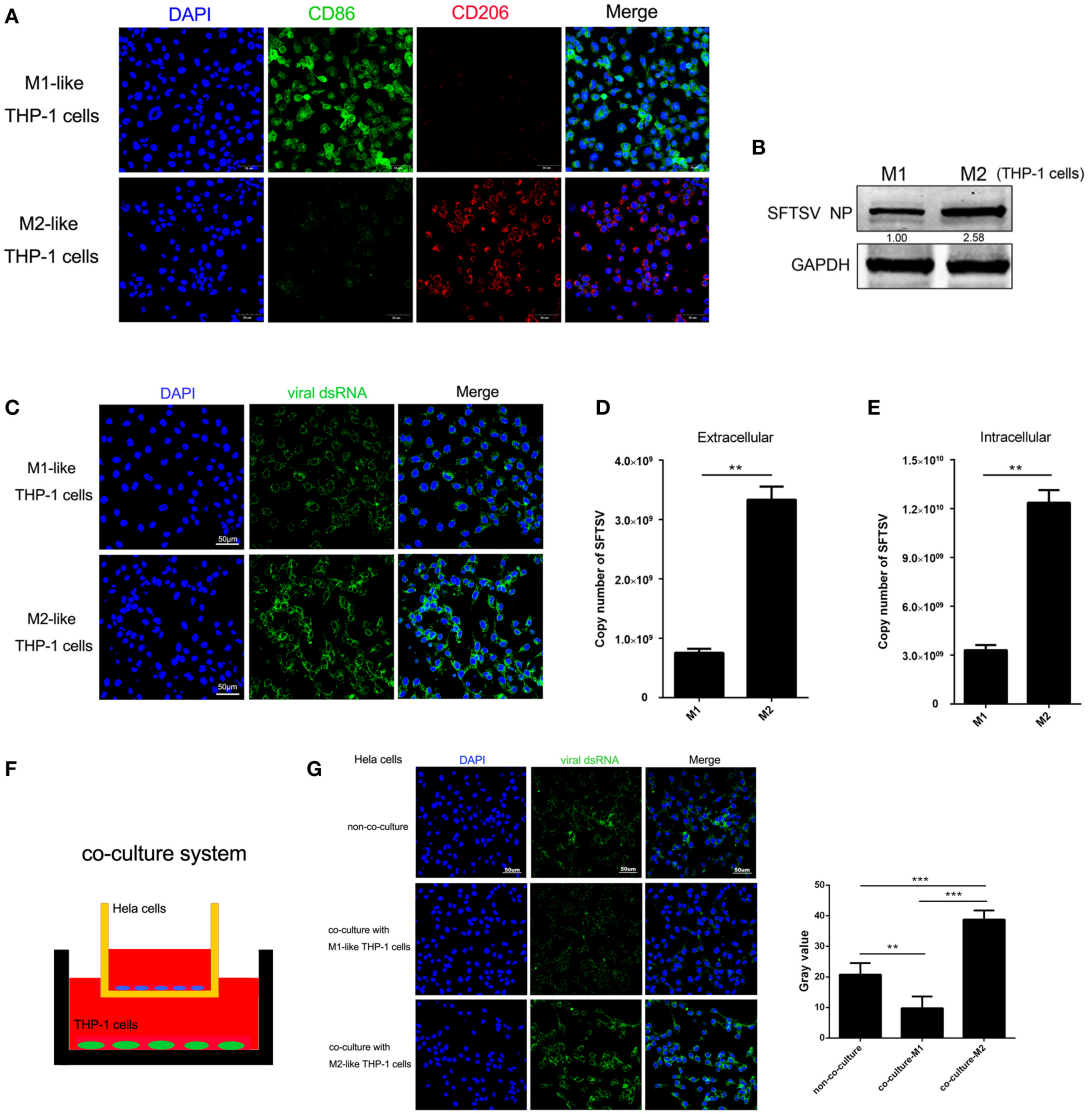
Macrophage differentiation affected SFTSV replication. **(A)** THP-1 cells are differentiated into M1 macrophages by 16 h incubation with 150 nM PMA and followed by incubating with IFN-γ and LPS, or into M2 with interleukin 4 and interleukin 13 for 24 h. Cells were then fixed and immunolabeled for CD86 or CD206 using specific antibodies. Nuclei were stained with DAPI (blue). Bar = 50 μm. **(B)** Western blot analysis of SFTSV nuclear protein (NP) in M1-like or M2-like cells after infection with SFTSV for 24 h at an MOI = 1. **(C)** Immunofluorescence staining of viral dsRNA (green) in M1-like or M2-like cells after 24 h infection. Nuclei were stained with DAPI (blue). Bar = 50 μm. **(D,E)** Quantitative real-time-PCR analysis of replicative copy number of SFTSV in the extracellular **(D)** or intracellular **(F)** compartments of the infected M1-like or M2-like cells. **(F)** Schematic presentation of the transwell co-culture with SFTSV-infected Hela cells in the top well and polarizing THP-1 cells in the bottom well. **(G)** Immunofluorescence staining of viral dsRNA (green) in Hela cells. Nuclei were stained with DAPI (blue). Bar = 50 μm. All data are presented as the mean ± SEM of three independent experiments. **(B,C)** (^**^P < 0.01, ^***^P < 0.001).

To determine whether macrophage differentiation influences the virus replication, THP-1 cells were induced to differentiate into macrophages by PMA. For M1 polarization, macrophages were cultured in the presence of IFN-γ in combination with LPS for 24 h while M2 macrophages were induced by IL-4 and IL-13 stimulation for 24 h, which were confirmed by immunofluorescence staining of CD86 and CD206, respectively (Figure 2A). We then analyzed SFTSV replication in the M1 or M2 macrophages and showed that SFTSV replicated higher in M2 than in M1, as shown by significantly higher expression of the SFTSV nuclear protein (NP) in M2 than in M1 macrophages (Figure 2B) and immunofluorescent staining of the intracellular viral double-strand RNA (dsRNA) (Figure 2C). To investigate the impact of macrophage differentiation on viral spread, we measured the intracellular and extracellular virus titers in M1 and M2 macrophages after a single cycle of viral infection. No cell death was observed for this infection (data not shown). As shown in Figures 2D,E, M2 macrophages showed significantly higher viral copy numbers in both extracellular and intracellular compartments than those in M1 macrophages, suggesting that SFTSV replicates more efficiently in M2 macrophages.

To examine whether the differential replication of SFTSV in the M2 and M1 macrophage-like THP-1 cells was mediated by soluble factors, we co-cultured the M1 or M2 macrophage-like THP-1 cells with SFTSV-infected Hela cells for 16 h in a trans-well system (Figure 2F). The intracellular viral dsRNA in Hela cells showed a higher staining in M2-Hela co-culture than that in M1-Hela co-culture (Figure 2G), suggesting that soluble factors from M2 macrophage-like THP-1 cells facilitated higher viral replication in Hela cells. In addition, the intracellular viral dsRNA in M1-Hela co-culture showed a significantly lower staining than SFTSV-infected Hela without co-culture, indicating that M1 secreted inhibitory factors IFN-γ that downregulated SFTSV replication.

### SFTSV Infection of THP-1 Upregulated miR-146

**Figure 3:**
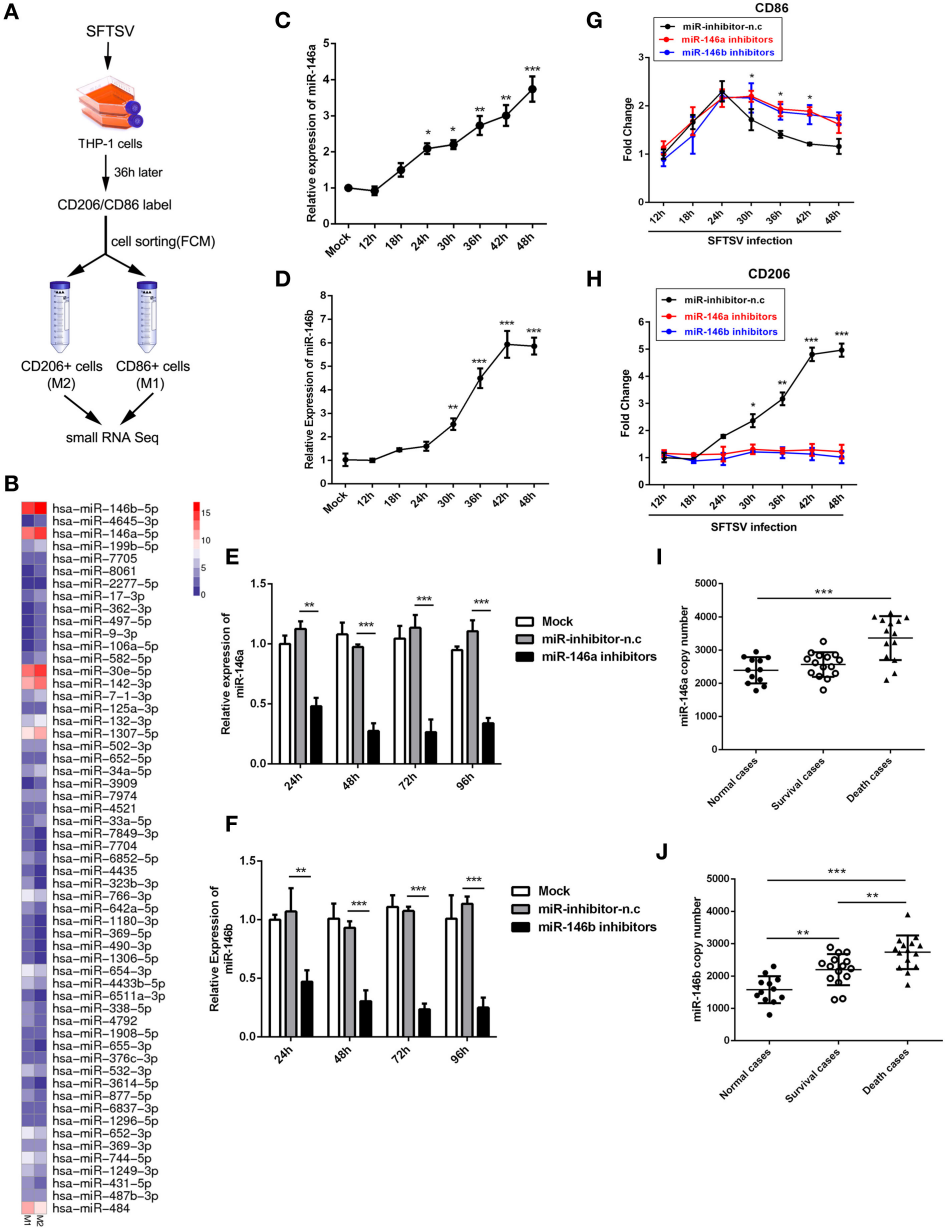
miR-146 modulated SFTSV induced macrophage differentiation. **(A)** Schematic presentation of SFTSV infected THP-1 cell sorting. THP-1 was infected with SFTSV for 36 h at an MOI of 1, adhered cells re-suspended, washed once in RPMI and surface stained and sorted following standard Biolegend intracellular flow cytometry staining protocol. M1 macrophages are CD11b^+^ CD86^+^CD206^−^ cells, whereas M2 macrophages are CD11b^+^ CD86^−^CD206^+^ cells. **(B)** Representative heatmap of M1 and M2 cells through deep miRNA sequencing. **(C,D)** Real-time PCR analysis of miR-146a **(C)** and b **(D)** expression level in THP-1 cells at various time points. **(E,F)** Real-time PCR analysis of miR-146a/b expression in THP-1 cells after being transfected with miR-146a/b inhibitors or negative control (miR-inhibitors-n.c) for various durations. **(G,H)** Real-time-PCR analysis of CD86 or CD206 expression on THP-1 cells infected with SFTSV before transfection of miR-146a or b at various time points. **(I,J)** Expression levels of miR-146a **(I)** and 146b **(J)** were measured by real-time PCR in SFTSV infected patient sera. All the data are shown as mean ± SEM of three independent experiments (^*^P < 0.05, ^**^P < 0.01, ^***^P < 0.001).

Accumulating evidences have shown that microRNAs could modulate macrophage differentiation (29). Therefore, we first investigated miRNA expression profiles in FCM sorted M1 and M2 macrophage-like THP-1 cells infected by SFTSV using small RNA sequence techniques (Figure 3A). As shown by RNA-seq analysis of M1- and M2-like macrophages in Figure 3B, 57 miRNAs exhibited differential expression with 11 increased by more than 1.5-fold and 15 decreased by more than 1.5-fold (Supplementary Table 1). Among these miRNAs, we focused on miR-146a/b that was maximally upregulated upon SFTSV infection and that it was previously reported to modulate macrophage differentiation by targeting various transcription factors and adaptor proteins (36, 37). We analyzed the miR-146a/b expression by quantitative real-time PCR and confirmed the up-regulation of the miRNA in SFTSV-infected THP-1 cells (Figures 3C,D). To determine whether the miR-146a/b were responsible for SFTSV-induced macrophage differentiation, we suppressed the endogenous miR-146a/b expression by transfecting THP-1 cells with specific inhibitors and analyzed the differentiation of macrophages induced by SFTSV by measuring the expression of CD86 or CD206 with qRT-PCR. As shown in Figures 3E,F, the inhibitors significantly inhibited the expression of the miR-146a/b miRNAs, which resulted in the up-regulation of CD86 at 30 h pi as compared to the negative control. In contrast, CD206 expression was down-regulated in cells treated with miR-146/b inhibitors as compared to the negative control. These results suggested that miR-146a and b drive SFTSV-induced THP-1 macrophage differentiation (Figures 3G,H). In addition, we evaluated the miR-146a/b levels in serum specimens of SFTSV-infected patients and found that both miR-146a and b copy numbers were significantly elevated in fatal cases (samples = 14) as compared with the uninfected controls (samples = 12). However, there were no significant differences for the miR-146a between the survival (samples = 15) and the uninfected controls (Figures 3I,J).

### miR-146a/b Targeted STAT1 and Drove THP-1 Differentiation During SFTSV Infection

**Figure 4:**
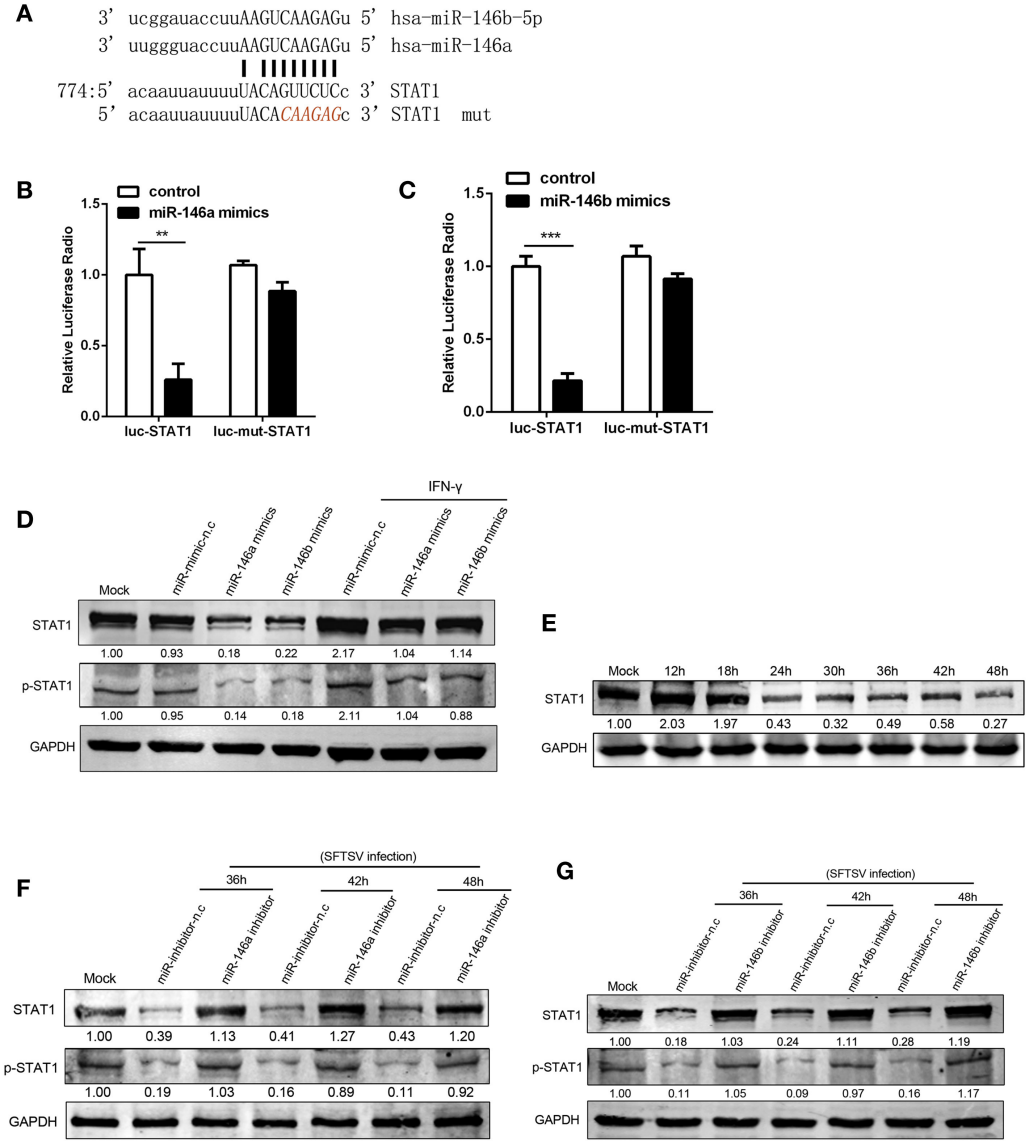
miR-146a/b targeted STAT1 and drove THP-1 differentiation during SFTSV infection. **(A)** Schematic diagram of the miR-146a/b target of STAT1. Shown is a sequence alignment of miR-146a/b and its target sites in 3′-UTR of STAT1. The mutations introduced by site-directed mutagenesis are shown (red). **(B,C)** The luciferase activity of STAT1 3′UTR is repressed by transfected miR-146a **(B)** or b **(C)** in 293T cells. Mutation of a predicted miR-146a/b binding site in the STAT1 3′UTR abolished miR-146a/b-mediated repression. **(D)** The expression of STAT1 and STAT1 phosphorylation is measured by western blot analysis in THP-1 cells transfected with miR-146a/b mimic, as well as the negative control (mimic-n.c) before treated with or without IFN-γ stimulation. The numbers denote the relative density of the bands normalized to the mock cells. Value of mock treatment is set at 1.00 (100%). **(E)** Western blot of STAT1 protein expression in SFTSV-infected THP-1 cells at various time points. **(F,G)** Western blot analysis of STAT1 and STAT1 phosphorylation in SFTSV infected cells before transfected with miR-146a **(F)**, miR-146b **(G)** inhibitor or inhibitor-n.c. ^**^P < 0.01, ^***^P < 0.001.

At post-transcriptional level miRNAs negatively regulate the expression of their target genes, through binding to complementary sequences at mRNA 3′UTRs (38). STAT1 is a critical molecule in IFN-γ signaling pathway and plays a crucial role in M1 activation (39, 40). STAT1 is a predicted target of miR-146 by a number of microRNA target databases (Figure 4A). Consistent with previous findings and database prediction, we found that miR-146a and b significantly reduced luciferase activity of a reporter gene containing the STAT1 3′UTR, and the deletion of 6 nt (Figure 4A) in the 3′UTR abolished the inhibitory effect of miR-146a or b, indicating that the observed down-regulation was dependent on the predicted miR-146a/b target sequence (Figures 4B,C). Consistent with this, THP-1 cells transfected with miR-146a/b mimics showed a significant reduction of STAT1 protein expression and phosphorylation as compared with THP-1 cells transfected with mimic negative control (mimic-n.c), which was completely reversed by IFN-γ treatment (Figures 4D,E). These data demonstrated that miR-146a/b targeted STAT1 and inhibited phosphorylation of STAT1. When endogenous miR-146a/b were inhibited, STAT1 expression was fully restored in response to SFTSV infection; in contrast STAT1 was reduced by 57–82% and phosphorylation of STAT1 was reduced by 81–91% depending on time of measurement in miR negative controls (Figures 4F,G), suggesting that SFTSV infection suppresses STAT1 expression by upregulating miR-146a/b.

### IL-10 Regulated miR-146b During SFTSV-Induced Macrophage Differentiation

**Figure 5:**
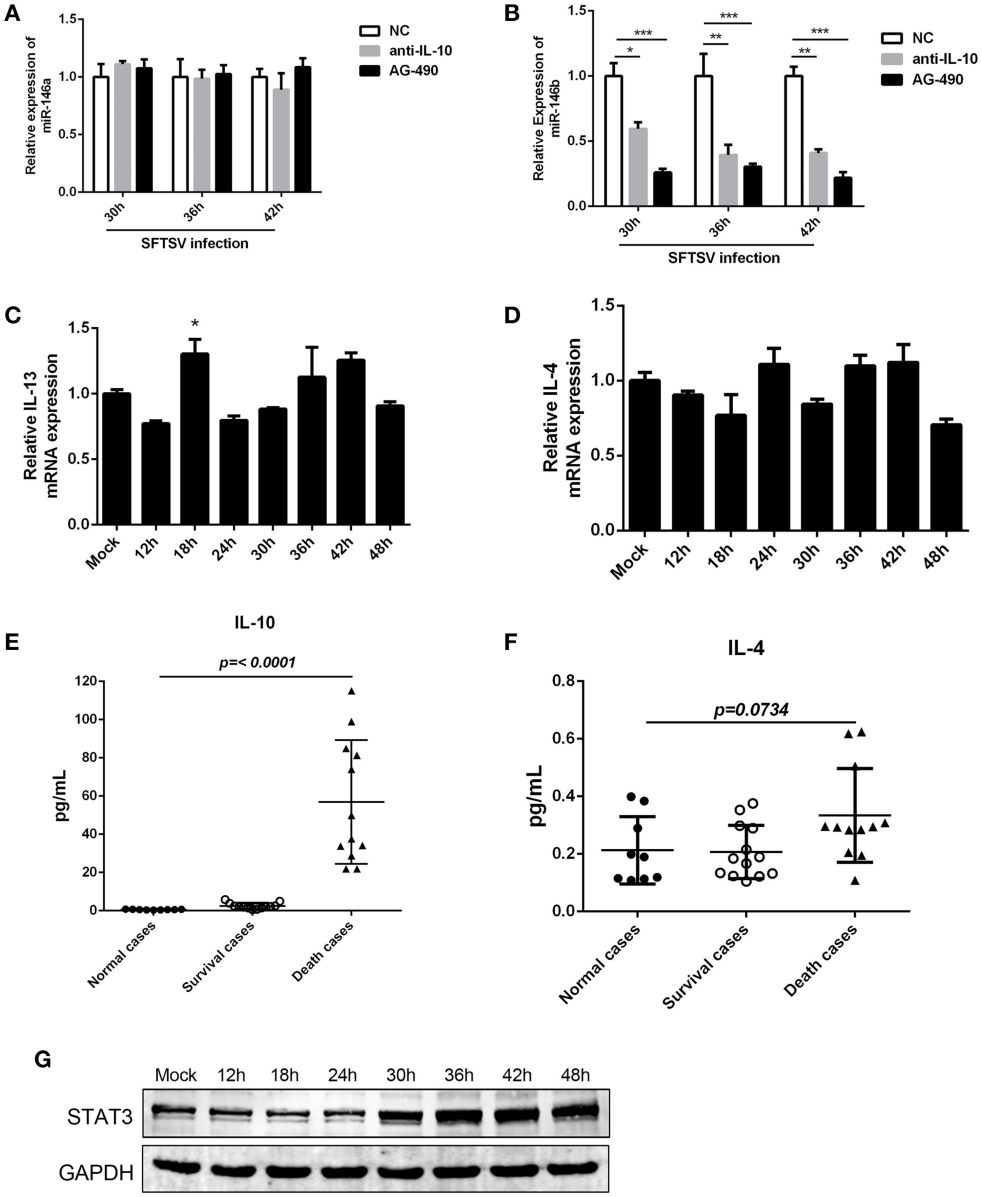
IL-10 induced the expression of miR-146b during SFTSV-induced macrophage differentiation. **(A, B)**The expression of miR-146a **(A)** or b **(B)** of THP-1 cells pretreated or not pretreated for 5 μM of the JAK/STAT inhibitor AG-490 or 10 μg/mL anti-IL-10 receptor (-αIL-10R) mAb and then infected with SFTSV at an MOI of 1 by quantitative real-time PCR. **(C,D)** Real-time PCR analysis of IL-13 **(C)** and IL-4 **(D)** mRNA expression in THP-1 cells infected with SFTSV. **(E,F)** Expression levels of IL-10 **(E)** and IL-4 **(F)** were measured using ELISA in SFTSV infected patient sera. **(G)** Western blot of STAT3 protein expression in SFTSV-infected THP-1 cells at various time points. Data are shown as means with ±SEM of three independent experiments (^*^P < 0.05, ^**^P < 0.01, ^***^P < 0.001).

The interplay of various cytokines plays a crucial role in the pathogenesis of SFTSV infection (41–43). Clinical studies have indicated that cytokine storm, characterized by the production of assortment of inflammatory cytokines, is associated with the disease severity (42, 43). We observed that IL-10, which is involved in modulation of miR-146 expression (44), was elevated in SFTS patients and produced at robust levels in fatal cases (42, 45). And notably, our initial study confirmed that IL-10 expression was upregulated during SFTSV infection of THP-1 cells (as shown in Figure 1B). To investigate the association between the miR-146 expression and IL-10 expression in SFTSV-infected THP-1 cells, we used an IL-10R specific neutralizing antibody or JAK/STAT inhibitor AG-490 and found that anti-IL-10R or AG-490 resulted in a significant reduction of SFTSV-induced miR-146b upregulation in the infected THP-1 cells, whereas miR-146a expression was not affected (Figures 5A,B). Previous study revealed that IL-4 and IL-13 could also induce the expression of miR-146b (37); however, our study showed that both IL-4 and IL-13 remained unchanged during SFTSV infection (Figures 5C,D). Furthermore, we analyzed the production of the cytokines in both the deceased and survived cases as well as in healthy donors and found that the IL-10 level in the deceased patients showed a more robust production than those in the survived and healthy cases, but the IL-4 level only slightly elevated in the deceased cases as compared to the healthy donors (Figures 5E,F). STAT3 protein expression did not show apparent change at 24 h after SFTSV infection, but showed significant increase from 30 hpi (Figure 5G). Together, these data indicate that miR-146a and b play distinct roles in regulating SFTSV-induced macrophage differentiation and identify miR-146b, but not miR-146a, as an IL-10-induced miRNA, suggesting that miR-146b may play a key role in mediating SFTSV-induced macrophage differentiation.

### Viral Non-Structural Protein (NSs) Contributed to miR-146b Expression

**Figure 6:**
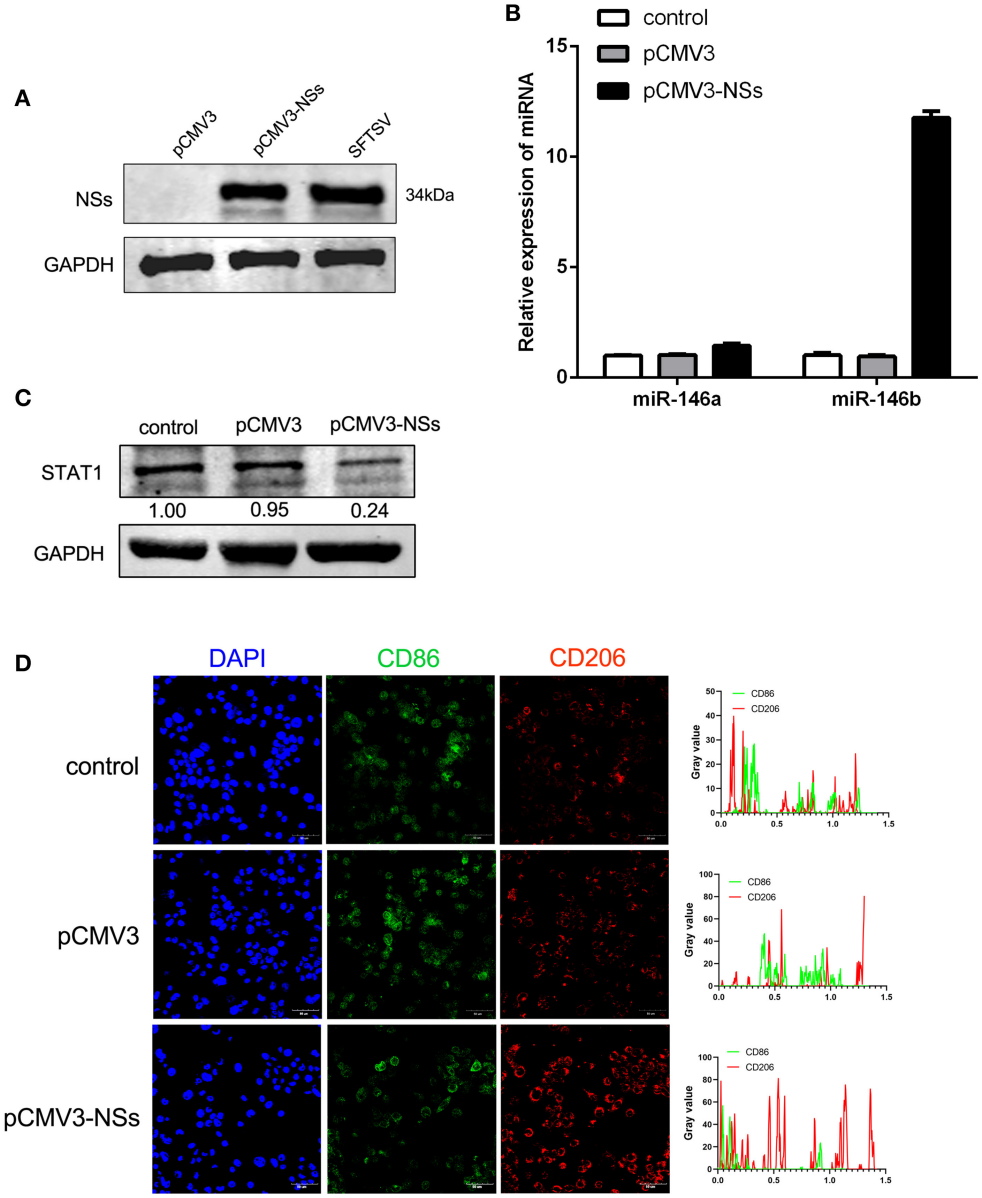
Viral non-structural protein (NSs) contribute to miR-146b expression resulted in inducing M2 macrophage differentiation. **(A)** Western blot analysis of SFTSV non-structural protein (NSs) and miR-146a/b expression **(B)** in THP-1 cells that were transfected control pCMV3 plasmid, NSs over-expression plasmid (pCMV3-NSs) for 48 h, respectively. SFTSV-infected THP-1 cells as a positive control. **(B,C)** THP-1 cells were transfected pCMV3 plasmid or pCMV3-NSs for 48 h. The expression of miR-146a/b and endogenous STAT1 detected by real-time PCR and western blot, respectively. **(D)** Immunofluorescence staining analyzed for CD86 and CD206 expression after transfected with pCMV3 or pCMV3-NSs for 48 h. Images were acquired using a confocal microscope. Right graph: fluorescence intensity of CD86 (green) and CD206 (red) in the regions delineated by a white line through ImageJ software.

To further explore the mechanism of miR-146 up-regulation upon SFTSV infection, we investigated the non-structural protein (NSs), which was reported to be involved in viral replication and modulation of host response (46). We first constructed NSs expression plasmid (pCMV3-NSs) and expressed the protein in THP-1 cells with pCMV3 plasmid as control (Figure 6A). Interestingly, NSs protein stimulated the expression of miR-146b but not miR-146a as shown by real-time PCR (Figure 6B). Moreover, transfection of THP-1 cells with pCMV3-NSs resulted in the suppression of endogenous STAT1 expression as compared to the cells transfected with pCMV3 control plasmid (Figure 6C). Immunofluorescence staining of CD86 and CD206 indicated that the cells transfected with pCMV3-NSs showed higher CD206 expression than the cells transfected with pCMV3 or the control (Figure 6D). These data suggest that endogenous miR-146b-induced differentiation of macrophages into M2 cells is mediated through NSs protein.

### SFTSV Induced Macrophage Differentiation *in vivo*

**Figure 7:**
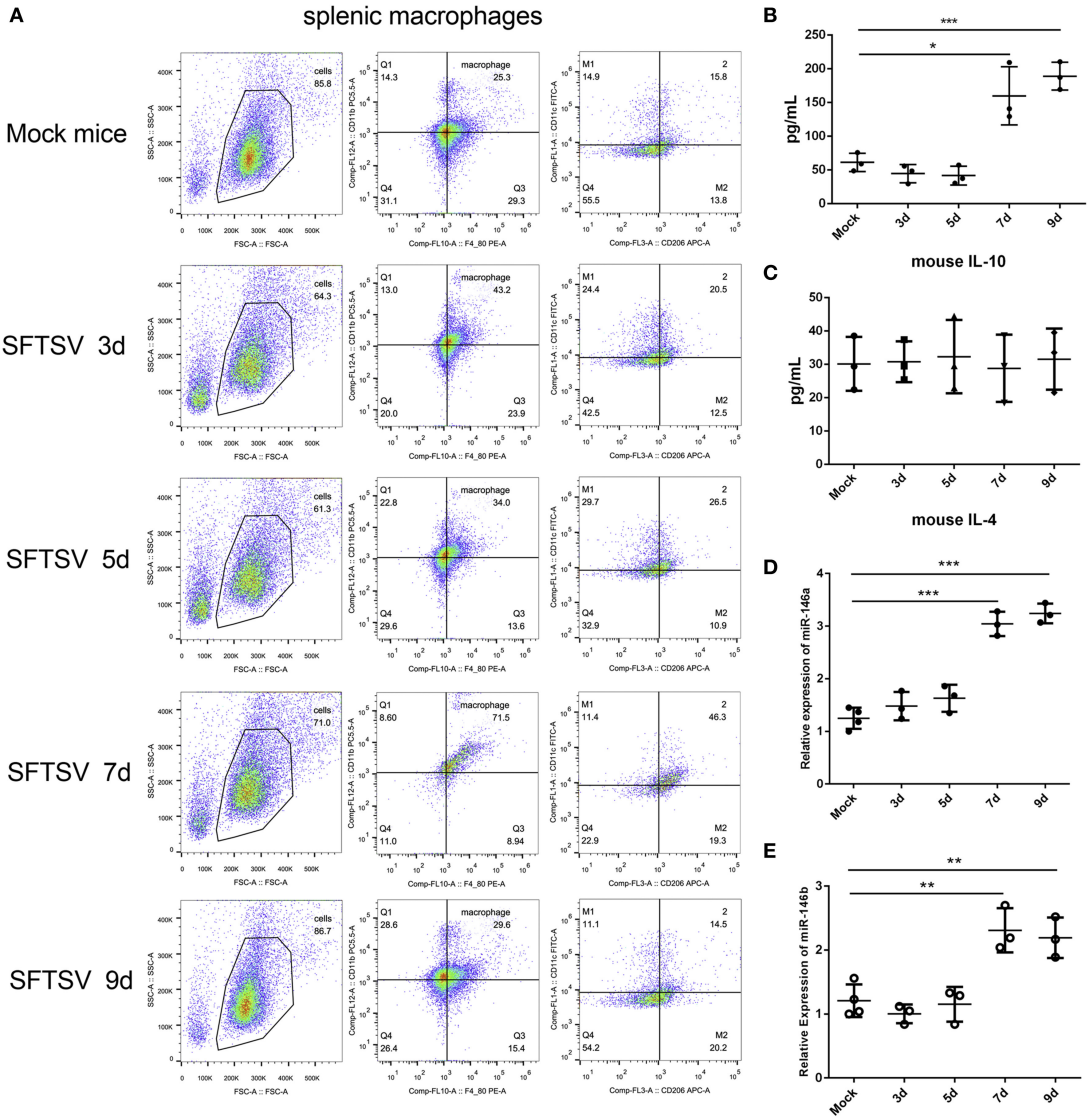
SFTSV induced macrophage differentiation *in vivo*. **(A)**. Flow-cytometry profiling of mouse splenic macrophages subsets after SFTSV infection. Macrophages were gated CD11b^+^ and F4/80^+^, M1 macrophages were gated CD11c^+^, and M2 macrophages were gated CD206^+^. **(B,C)** The expression level of IL-10 **(B)** and IL-4 **(C)** were determined by ELISA in mouse serum that infected with SFTSV. **(D,E)**The expression of miR-146a **(D)** and miR-146b **(E)** within sorted mouse splenic macrophages in each group (*n* = 3). Data in all panels are shown as mean ± SEM (^*^P < 0.05, ^**^P < 0.01, ^***^P < 0.001).

SFTSV infection is characterized by drastic failure of blood clotting ability and the reduction of white blood cells, which was reproduced in C57/BL6 mice (6). Therefore, we used C57/BL6 mice as a model to investigate the *in vivo* roles of macrophages in SFTSV infection. A group of 12 mice were infected by intramuscular inoculation of 10^5^ TCID_50_ SFTSV per mouse and the mice were sacrificed on days 3, 5, 7, and 9. A group of three mice mock-infected served as control. Spleens from the infected mice exhibited splenomegaly and the analysis of the splenic macrophage phenotypes showed significantly increased expression of M1 marker CD86 between day 1–5 pi and decreased gradually from day 7 pi. However, the expression of the M2 marker, CD206, was dramatically elevated from day 7 pi (Figure 7A), indicating that SFTSV infection induced splenic macrophage differentiation to M2 phenotype at a later stage of infection in C57/BL6 mice. Analysis of serum cytokine levels of the infected mice showed that the IL-10 expression increased along with the increased expression M2 marker CD206 from day 7 pi, while IL-4 remained unchanged (Figures 7B,C), consistent with the *in vitro* results. In addition, the expression of miR-146a/b in mouse spleen macrophages was significantly elevated after 7 days (Figures 7D,E).

### SFTSV Reduced MHC-II and CD86 Expression on Macrophages

**Figure 8:**
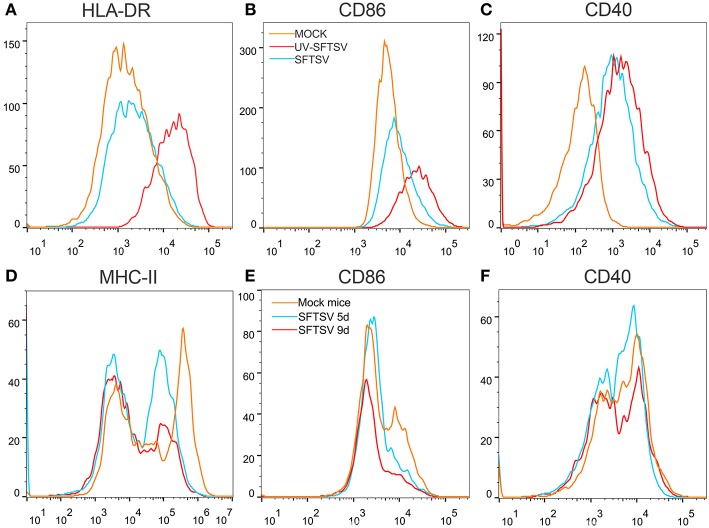
SFTSV suppresses MHC-II and CD86 expression on activated macrophage. **(A–C)** THP-1 cells were infected with SFTSV UV-inactivated SFTSV or mock-infected at an MOI = 1. After non-specific binding being blocked by human TruStain FcX™ (BioLegend, USA), CD11b^+^ macrophages were analyzed for cell surface expression of HLA-DR **(A)**, CD86 **(B)**, and CD40 **(C)** by FACS using the indicated antibodies: Mock (orange line), UV-inactivated SFTSV (red line), or SFTSV (blue line). **(D–F)** Four-week-old male C57/BL6 mice (*n* = 4) were inoculated with 10^5^ TCID_50_ SFTSV per mouse through intravenous injection and four mock-infected mice as controls. After non-specific binding being blocked by mouse Fc Block™ (BD Biosciences, USA), mouse splenic CD11b^+^ F4/80^+^ macrophages were analyzed for cell surface expression of MHC-II **(D)**, CD86 **(E)**, and CD40 **(F)** by FACS using the indicated antibodies: Mock (orange line), 5d SFTSV (blue line), or 9d SFTSV (red line).

To investigate the mechanism by which SFTSV suppressed macrophage activation of CD4 T cells, THP-1 cells were infected with SFTSV, UV-inactivated SFTSV or not infected at an MOI of 1. As expected, UV-inactivated SFTSV greatly enhanced the expression of co-stimulatory molecules of HLA-DR, CD86, and CD40 on the macrophages. SFTSV-infected macrophages showed the similar level of CD40 as that of UV-inactivated SFTSV (Figure 8C) but the expression of both HLA-DR and CD86 was inhibited (Figures 8A,B), consistent with the temporal expression of CD86 (Figure 1A) and the observation in patients (47). SFTSV infection of activated macrophages resulted in the down-regulation of surface HLA-DR and CD86 but not CD40. To investigate whether SFTSV influences mouse splenic macrophages co-stimulatory molecules expression, a group of eight C57/BL6 mice were infected by intramuscular inoculation of 10^5^ TCID_50_ SFTSV per mouse and the mice were sacrificed on days 5 and 9. A group of three mice mock-infected served as control. Macrophages from SFTSV-infected mice showed the down-regulated surface expression of MHC-II and CD86, which showed further downregulation as the infection proceeded (Figures 8D,E), as compared with the mock-infected mice. Interestingly, CD40 was not affected by infection with either SFTSV or UV-inactivated SFTSV (Figure 8F).

## Discussion

**Figure 9:**
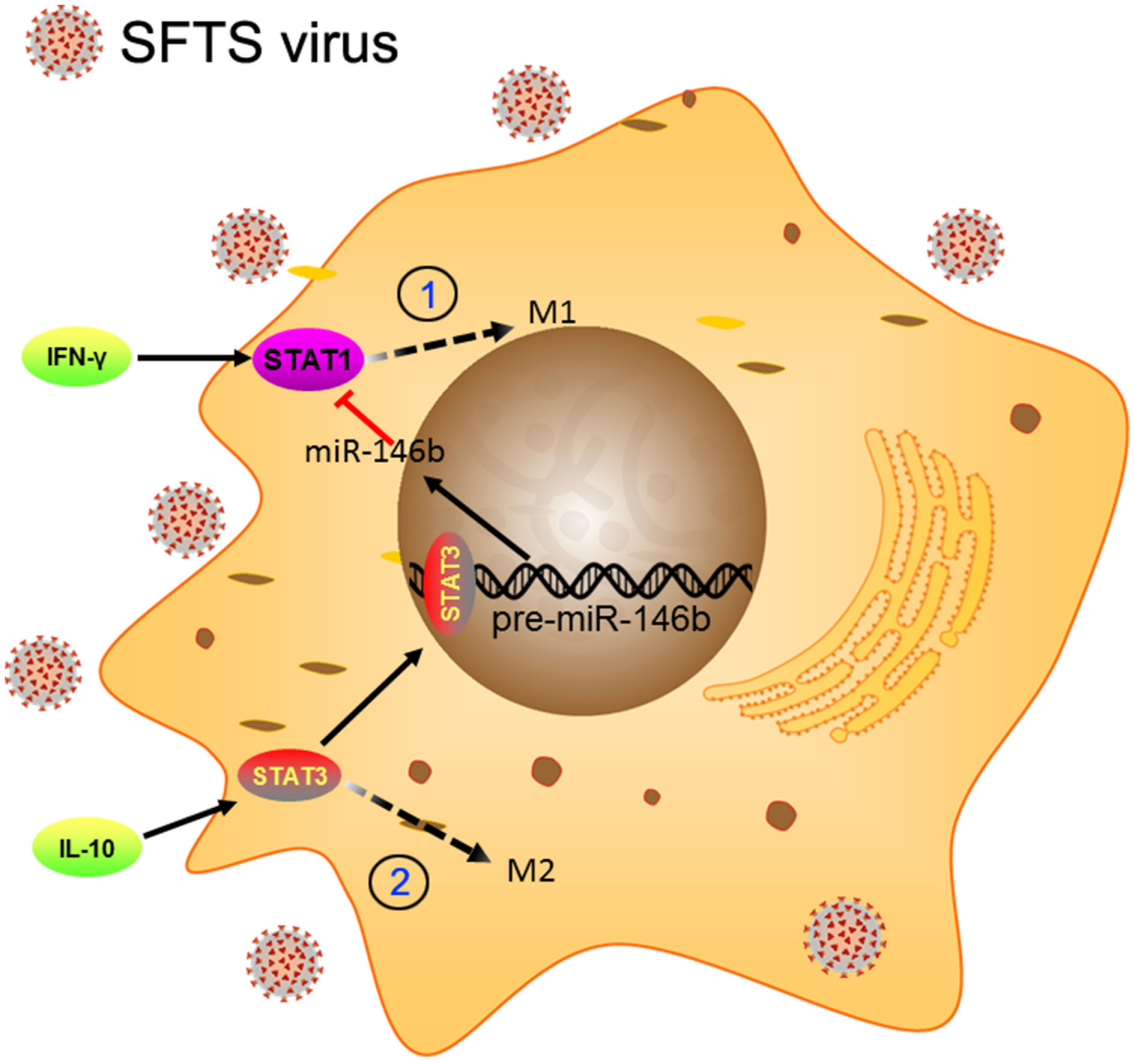
Schematic diagram showing the roles of miR-146b in the regulation of activation of macrophages during SFTSV infection. At the early stage of infection, the SFTSV induced monocyte immune response and stimulates the differentiation of macrophages to M1 cells through activation of STAT1. When SFTSV begins to replicate, the host immune response switches from M1 to M2 type and upregulates IL-10 that stimulates differentiation of macrophages to M2 cells through activation of STAT3. Meanwhile, IL-10 but not IL-4/13 induces the expression of miR-146b by activating STAT3, which binds to the promoter of the pre-miR-146b gene and initiates transcription. The resulting miR-146b inhibits the differentiation of M1 macrophages by targeting STAT1 and driving macrophages to M2 phenotype.

Macrophages are known as an important component of the immune system, which is a critical first defense in innate immunity and modulates adaptive immune response to pathogens through antigen processing and presentation (8, 48). Essential to such functions is classical activation (M1) and alternative activation (M2) of macrophages (49). Previous studies have demonstrated that SFTS virus is capable of hijacking splenic macrophage for replication which was identified as primary target cells of SFTSV infection and the virus could harbor within splenic macrophages for long periods (6). A similar result was observed in an *in vitro* cell culture system using monocytic THP-1 cells (37). These studies imply that monocytes/macrophages may support persistent SFTSV infection. However, the role of macrophages in virus replication and the potential pathogenic mechanisms of SFTSV in macrophage remain unclear. In this study, we present evidence that SFTSV infection drove macrophage differentiation skewed toward M2 phenotype, which was modulated by miR-146a/b that was significantly upregulated in macrophages by SFTSV infection. Further evidence showed that the SFTSV-induced upregulation of IL-10 upregulated the expression of miR-146b but not miR-146a and viral NSs is a viral component that mediated the increased miR-146 expression (Figure 9).

SFTSV infection drove the THP-1 differentiation skewed toward M2 macrophage as evidenced by continuously increased expression of M2 markers, such as IL-10, CD163, CD206, and CCL22 (50). The initial increase of M1 in the 18–24 hpi may be explained by the fact that THP-1 is a monocytic cell line and the M2 differentiation will need to come from activated macrophages instead of directly from monocytes. CD86 and CD206 are co-expressed on undifferentiated THP-1 cells. As the cells are infected by SFTSV, CD86 expression decreases and CD206 increases as the infection proceeds from 24 to 48 h, as evidenced by both flow cytometric analysis and IF staining. Earlier studies demonstrated that certain stimuli could drive the switch between M1 and M2 (51). Indeed, evidence showed that M2 phenotype tends to appear later during macrophage differentiation (12). Distinct functions of macrophages are associated with the type of receptor interaction and the presence of cytokines (8). The classical M1 macrophages are activated by “pro-inflammatory” cytokines (i.e., IL-6 and TNF-α) and act to kill intracellular pathogens, while the alternative M2 macrophages appear to be involved in immunosuppression (8, 49, 52). IL-4 and IL-13, and also IL-10 and TGF-β, induce alternatively activated M2 phenotype polarization, which are poor APCs and are suppressors of Th1 responses (53). M1 function is pro-inflammatory, M2a is anti-inflammatory induced by IL-4 and IL-13 (51). We suspect whether the virus inhibits inflammation is beneficial to its own replication, therefore, we focused on the roles of the M2a macrophages. We suggest that the M2 skewed macrophage differentiation is a strategy for virus to evade clearance by host antiviral immune response such as Th1 cytokines which are produced by M1 macrophages. For instance, TNF-α triggers local containment of infection, and IL-6, as well as IL-1β, could reduce viral replication or increase antigen processing, thus promoting specific immune response (54). Indeed, our study showed that the SFTSV N protein (NP) synthesis was more efficient in M2 macrophages than in M1 macrophages. The N protein of SFTSV is associated with replication products throughout the infectious life cycle, and, by binding to viral genome to form a ribonucleoprotein (RNP) complex, NP plays a critical role in virus viability (55, 56). Together, our data suggest that the SFTSV drives macrophages differentiated toward M2 phenotype to facilitate viral replication.

miRNAs have been shown to play roles in macrophage differentiation. For instance, miR-9, miR-127, miR-155, and miR-125b were shown to promote M1 polarization while miR-124, miR-223, miR-34a, let-7c, miR-132, miR-146a, and miR-125a-5p induced M2 polarization by targeting various transcription factors and adaptor proteins (25, 31, 57). Hence, miRNAs that modulate macrophage polarization are considered to have therapeutic potential in the treatment of inflammation-related diseases (31). Our data suggest that miR-146a and b play important roles in THP-1 macrophage differentiation to M2 phenotype upon SFTSV infection. The increase of miR-146a/b were appeared from 24 hpi could explains the initial M1 increase of M1 and subsequent M2 increase with corresponding decrease of M1. In view of the regulatory roles of IL-10 on miR-146b, the dynamic temporal changes of M1 and M2 phenotypes were corresponding to the changes of cytokines. Further experiments confirmed that both miR-146a and b acted by directly targeting STAT1, a pivotal molecule in IFN-γ signal transduction. miR-146a and b share the same seed sequence and only differ by two nucleotides at the 3' end in their mature sequences, suggesting that they repress the same target genes and control similar biological processes (44, 58). Indeed, previous studies defined miR-146 as a negative regulator of inflammation, particular in virus invasion (44, 59–61). For example, the IRAK1 and TRAF6, two key adaptor molecules in the Toll like receptor and NF-κB pathway, are direct targets of miR-146 (59, 60, 62). Taganov et al. first reported that miR-146 modulates macrophage function and identified this miRNA as a regulator of classical NF-κB activation (63). Several studies have revealed that miR-146a directly inhibits the activation of M1 macrophage and plays an important role in the pathogenesis of human diseases, such as *Brugia malayi* infection (64), mycobacterial exposure (65), and nephropathy (66). In addition, He *et al* reported that both miR-146a and b were involved in the regulation of macrophage activation and have a potential for treating schistosomiasis through regulating differentiation of macrophages (37), further demonstrating that only miR-146b could be induced by a series of Th2 cytokines to inhibit the differentiation of macrophages to M1 cells through targeting STAT1. Our study expanded the understanding of miR-146 function in the macrophage differentiation upon the acute viral infection.

SFTS viral genome is composed of the large (L), medium (M), and small (S) RNA segments. The S segment encodes the nucleocapsid (N) protein and a non-structural protein (NSs). Among bunyaviruses, the non-structural protein NSs has been found to be a major virulent factor acting as a global inhibitor of host cell transcription and antagonist of the IFN system (67–69). For instance, expression of NSs resulted in inhibiting IFN-α-stimulated tyrosine phosphorylation and nuclear translocation of STAT2; in contrast, NSs affected neither subcellular distribution nor phosphorylation of STAT1 in response to IFN-α and IFN-γ (70). In addition, SFTSV infection promoted LC3 accumulation and NSs was co-localized with several proteins of the autophagy pathway (71). Our study showed NSs protein stimulated the expression of miR-146b, resulting in the inhibition of endogenous STAT1 expression and facilitating macrophage differentiation to M2 phenotype. It is plausible that NSs may suppress IL-10 to impact on miR-146b though this remains to be demonstrated.

It has been known that over-activation of immune system and the overproduction of inflammatory cytokines such as IL-6, IL-10, TNF-α, and monocyte chemoattractant protein-1 (MCP)-1 can create a “cytokine storm”, which is considered to contribute to the pathology of SFTS (43, 72). We observed that cytokine IL-10 could modulate miR-146b expression, consistent with an earlier report by Curtale et al (44). We reported that robust IL-10 expression was detected in the first week of STFTV infection in the deceased patients, in contrast to a significantly lower level in the convalescent patients (47), suggesting a causative relation between elevated IL-10 and miR-146a/b and the pathogenesis of SFTSV infection. Although miR-146a and b belong to the same family and share the same seed sequence, they are encoded by distinct genes located on separate chromosomes, which implies that they may fulfill distinct functions (73, 74), which is reflected by their distinct response to IL-10 stimulation Earlier studies have been mostly focused on the biological activities of miR-146a with respect to immune response, but little research was done on the miR-146b except that miR-146b was involved in the IL-10-dependent resolution phase of inflammation (44). In IL-10 stimulated macrophages, STAT3 was recruited to the pre-miR-146b (44). Through blocking the IL-10 signaling pathway, we found that only miR-146b but not miR-146a could be induced by IL-10 cytokine but not by IL-4 and IL-13 as previously reported (37).

Most tissue macrophages express class II MHC on their surface, it is further inducible by T cell products (notably IFN-γ) and is expressed at high levels on the macrophages recruited in response to an immune stimulus. However, it is reasonable that virus-induced M2 skewed macrophage differentiation will suppress the host immune response, reducing IFN-γ production, and increasing IL-10 production. IL-10 signaling also promotes expression of the microRNAs miR-146b that ultimately leads to the suppression of M1 macrophages (44). IL-10 has been reported to downregulate HLA-DR and suppress co-stimulatory molecules such as CD86 and CD40 (75). Our and other studies (47) in patients suggest that SFTSV suppresses macrophage antigen presentation through inhibition of co-stimulatory molecules to inactivate CD4 T cells, which mitigates the T cell ability to clear pathogens. It is a plausible strategy that SFTSV upregulates IL-10 to drive macrophage differentiation to M2 phenotype via miR-146b and in the meantime to inhibit the antigen presentation and co-stimulatory molecules to suppress adaptive immunity.

## Materials and Methods

### Cell Culture and Macrophage Polarization

Human monocyte cell line (THP-1) was purchased from Cell Resource Center of Shanghai Institute for Biological Sciences, Chinese Academy of Sciences, China. Human cervical carcinoma cell line (Hela) (ATCC, USA) was maintained in DMEM (Gibco, USA) supplemented with 10% (v/v) FBS (Gibco, USA), 100 μg/ml streptomycin, 100 U/ml penicillin at 37°C and an atmosphere of 5% CO_2_. THP-1 cells were differentiated in RPMI-1640 (Gibco, USA) medium supplemented with 10% FBS containing 5 mM L-glutamine, penicillin/streptomycin, and Phorbol-12-myristate-13-acetate (PMA 50 nM,Sigma, CA) for 24 h followed by culturing in fresh medium for another 24 h. M1 macrophages were generated by treatment of THP-1 cells with LPS (10 pg/ml, Sigma, GER) and IFN-γ (10 ng/ml, Sigma, GER) for 12 h. M2 macrophages were generated by treatment of the cells with IL-4 (20 ng/ml, Novus, USA) and IL-13 (20 ng/ml, Novus, USA) for 48 h.

### Transient Transfection of miR-146a/b Mimic and miR-146a/b Inhibitor

To overexpress or downregulate the expression of endogenous miR-146a/b, macrophages were transfected with miR-146a/b mimics (a chemically synthesized double-stranded RNA for overexpression) or an antago-miR-146a/b as inhibitors (RiBoBio, Guangzhou, China) using LipoGene^TM^ 2000 star transfection reagent (US Everbright, China) according to the manufacturer's protocol. Macrophages were plated in DMEM supplemented with 10% FBS at a density of 2–3 × 10^5^ cells/ml and were transfected with 100 nM miR-146a/b mimics, an antago-miR-146a/b, or a control miRNA mimic, respectively. After 6 h, the transfection medium was replaced with culture medium containing 10% FBS, and the cells were maintained at 37°Cin a 5% CO_2_ for 24 h, followed by infection with SFTSV (MOI = 1). The sequences of the oligonucleotides used are as follows: miR-146a mimic, forward, 5′-UGA GAA CUG AAU UCC AUG GGU U-3′ and reverse, 5′-CCC AUG GAA UUC AGU UCU CAU U-3′; miR-146b mimic, forward, 5′- UGA GAA CUG AAU UCC AUA GGC UG−3′ and reverse, 5′- CAG CCU AUG GAA UUC AGU UCU CA−3′; miR-NC mimic, forward, 5′-UUC UCC GAA CGU GUC ACG UTT-3′ and reverse, 5′-ACG UGA CAC GUU CGG AGA ATT-3′; antago-miR-146a, 5′-AA CCC AUG GAA UUC AGU UCU CA-3′; antago-miR-146b, 5′-CAG CCU AUG GAA UUC AGU UCU CA-3′; miR-NS inhibitor, 5′-UCU ACU CUU UCU AGG AGG UUG UGA-3′.

### RNA Isolation and Quantitative Real-Time PCR

Total RNA was isolated from THP-1 cells using TRIzol reagents (Invitrogen, USA), and reverse transcription of first-strand cDNA was performed at 37°C for 15 min, 85°C for 5 s, and 37°C for 10 min using a PrimeScript®RT reagent kit (Takara, Japan) according to the manufacturer's protocol. Quantitative real-time-PCR analyses for mRNAs of TNF-α, IL-1β, IL-6, CD86, IL-10, CD163, CD206, CCL22, and GAPDH were performed by using ABI SYBR Green Master Mix (Life Technologies) on ABI Prism 7,300 Sequence Detection System. The GAPDH mRNA was used as an internal control. The sequences of the oligonucleotides used are as follows: GAPDH, forward, 5′-GTC TTC ACC ACC ATG GAG−3′ and reverse, 5′-CCA AAG TTG TCA TGG ATG ACC-3; TNF-α, forward, 5′- CCCAGGGACCTCTCTCTAATCA-3′ and reverse, 5′- GCT ACA GGC TTG TCA CTC GG-3′; IL-1β, forward, 5′- AAT CTG TAC CTG TCC TGC GTG TT−3′ and reverse, 5′- TGG GTA ATT TTT GGG ATC TAC ACT CT−3′; IL-6, forward, 5′-AGT GCC TCT TTG CTG CTT TCA C-3′ and reverse, 5′-TGA CAA ACA AAT TCG GTA CAT CCT-3′; CD86, forward, 5′- TGG TGC TGC TCC TCT GAA GAT TC-3′ and reverse, 5′- ATC ATT CCT GTG GGC TTT TTG TG-3′; IL-10, forward, 5′-GCT CTT ACT GAC TGG CAT GAG-3′ and reverse, 5′-CGC AGC TCT AGG AGC ATG TG-3′; CD163, forward, 5′-TTG CCA GCT TAA ATG TG-3′ and reverse, 5′-AGG ACA GTG TTT GGG ACT GG-3′; CD206, forward, 5′-CGG TGA CCT CAC AAG TAT CCA CAC-3′ and reverse, 5′-TTC ATC ACC ACA CAA TCC TCC TGT−3′; CCL22, forward, 5′- GTT GTC CTC GTC CTC CTT GC-3′ and reverse, 5′- GGA GTC TGA GGT CCA GTA GAA GTG-3′; PCR was performed at 94°C for 5 min, followed by 30–35 cycles of amplification at 94°C for 40 s, 51°C for 40 s, and 72°C for 1 min by using ABI7500.

### Western Blotting

Cell lysates were prepared in standard RIPA buffer (Santa Cruz, USA) and cleared by centrifugation. Total proteins were quantified by BCA protein assay kit (Life Technologies, USA) and separated on 10% SDS-polyacrylamide gel, and transferred onto PVDF membranes (Millipore, USA). Proteins were detected with respective antibodies at 4°C overnight, followed by incubation with either IRDye Fluor 680-labeled IgG (1:10000 926-68071) or IRDye Fluor 800-labeled IgG (1:10000 926-32210) secondary antibody (Li-COR Bioscience). The images were scanned and quantified by densitometric analysis on Li-COR Odyssey Infrared Imager. Rabbit anti-STAT1 (1:1000 10144-2-AP) and STAT3 (1:1000 10253-2-AP) and mouse anti-GAPDH (1:1000 60004-1-Ig) antibodies were from Proteintech (China). Rabbit anti-phospho-STAT1 (Ser727) mAb (1:1000 D3B7) was purchased from Cell Signaling Technology, USA. Rabbit anti-SFTSV NP antibody (1:500 01-05-0130) was from Cambridge Bio (UK). Rabbit anti-SFTSV NSs antibody (1:1000 E7914) was custom ordered from ABclonal (China).

### ELISA

The concentrations of IL-4 and IL-10 in the mouse and human serum were measured by enzyme-linked immunosorbent assay (ELISA) kits (Elabscience Biotechnology Ltd., Co, China) according to the manufacturer's instructions. Replicate of each sample was measured and three independent experiments were performed. Optical density was measured at 450 nm.

### Flow Cytometry

THP-1 cells were trypsinized and re-suspended in ice-cold flow cytometry buffer (2% [v/v] FBS and 2 mM EDTA in PBS). After non-specific binding being blocked by human TruStain fcX™ (BioLegend, USA), M1 macrophages were identified by anti-human CD86-AF488 and M2 macrophages by anti-human CD206-APC (BioLegend, USA). Phenotypic analysis of M1 and M2 macrophages was performed on a flow cytometer (Verse, BD Bioscience). The data acquired were analyzed with FlowJo (Treestar software, Ashland, OR, USA). Mouse spleen was lysed in red blood cell lysis solution (Miltenyi Biotec, GER), washed once and re-suspended in flow cytometry buffer. After non-specific binding being blocked by mouse Fc Block™ (BD Biosciences, USA), the cells were incubated with anti-mouse F4/80-PE and CD11b-Percp-cy5.5 (BioLegend, USA) for 30 min at 4°C, following standard Biolegend intracellular flow cytometry staining protocol. Intracellular staining was carried out with anti-mouse CD206-AF647 (BioLegend, USA) on cell fixed at room temperature for 20 min with fixation buffer and permeabilized with intracellular staining permeabilization wash buffer (BioLegend, USA). M1 macrophages were identified using anti-mouse CD11c-AF488 and M2 macrophages by anti-mouse CD206-AF647 (BioLegend, USA). Flow cytometry events were gated based on forward and side scatter, and F4/80^+^ and CD11b^+^ cells were then selected for the analysis of the M1/M2 marker. Data were acquired on a CytoFLEX cytometer (Beckman, USA) and analyzed on FlowJo software (Tree Star, Ashland, OR).

### Cell Sorting

THP-1 was infected with SFTSV for 48 h, and the adhered cells were re-suspended, washed in RPMI, and surface stained following standard Biolegend intracellular flow cytometry staining protocol, and then processed for cell sorting. Mouse spleenocytes were lysed in red blood cell lysis solution (Miltenyi Biotec, GER), washed once in RPMI, and then processed for cell staining and sorting on BD FACS Aria II following standard protocol.

### SFTS Viral Load Assay

Serum viral RNA was extracted from patient blood (1 ml) using a viral RNA kit from QIAamp DSP Virus Spin Kit (Qiagen, GER) following the manufacturer's procedures. Total viral RNA was extracted from 0.5 × 10^6^ M1 or M2 macrophages intracellular viral RNA or from culture media as extracellular viral RNA using a commercial kit from QIAamp DSP Virus Spin Kit (Qiagen, GER) following the manufacturer's procedures. The SFTSV viral load was determined by one-step real-time RT-PCR using QuantiTect Probe RT-PCR kit (Qiagen, GER). A plasmid containing the full length of the SFTSV S segment was used as a standard for calculation of the SFTSV genome copies. The sequences of oligonucleotides used are as follows: SFTSV S segment, forward, 5′- TGC CTT CAC CAA GAC TAT CAA TGT−3′ and reverse, 5′-GGG TCC CTG AAG GAG TTG TAA A-3′, probe 5'-FAM-TTC TGT CTT GCT GGC TCC GCG C-BHQ1-3′.

### Immunofluorescence

Macrophages were fixed with 4% paraformaldehyde for 10 min, permeabilized with 0.1% Triton™ X-100 for 10 min, and blocked with 1% BSA for 1 h. The cells were then incubated with primary antibodies: rabbit anti-human CD86 (ab53004, Abcam, Cambridge, UK) at 1:500 dilution or mouse anti-human CD206 (170710, Bio-Rad, USA) at 1:200 dilution overnight at 4°C. The double-stranded RNA (dsRNA) was stained with anti-dsRNA monoclonal antibody (English & Scientific Consulting Kft.; K1-1301) at 1:2,000 dilution. The secondary antibodies Alexa Fluor 488 goat anti-rabbit IgG (H + L) (A-11034, Invitrogen, USA) or Alexa Fluor 594 goat anti-mouse IgG (H + L) (A11032, Invitrogen, USA) were used at 1:1,000 dilution for 1 h. 4′,6-Diamidino-2-Phenylindole (DAPI) was used to stain the nucleus at a concentration of 100 ng/ml.

### Animal Experiment

All animal experimental protocols were approved by the Nanjing University Animal Care Committee and followed the “Guide for the Care and Use of Laboratory Animals” published by the Chinese National Institutes of Health. Four-week-old male C57/BL6 mice (*n* = 5) were inoculated with 10^5^ TCID_50_ SFTSV per mouse through intravenous injections and four mock mice were used in parallel as controls. At each time point, the mice were sacrificed for tissue sections. The mouse spleenocytes were lyzed in red blood cell lysis solution (Miltenyi Biotec, GER), washed once in RPMI, and then processed for cell staining and splenic macrophage sorting following standard FACS protocol. miR-146a and b in splenic macrophages were analyzed by real-time RT-PCR. Serum IL-10 and IL-4 were detected by commercial ELISA kits (R&D, USA).

### Small RNA Deep Sequencing Analysis

THP-1 cell was infected with SFTSV for 36 h, and the adhered cells were re-suspended and washed once in RPMI, then processed for cell staining and sorting on BD FACS Aria II to separate M1 and M2 macrophages. Deep sequencing of the miRNA profiles was performed by Annoroad Genomics with the Illumina Hiseq 2,500 platform with three different sample preparations.

### Clinical Samples

Clinical samples were collected from confirmed SFTS patients at Nanjing Drum Tower Hospital, as described in our previously published article (76). All experiments were performed in accordance with relevant guidelines and regulations.

### Statistical Analysis

Statistical analysis was performed using SPSS16.0 statistical software. Values were expressed as mean ± SEM. One-way analysis of variance was performed for multiple group comparisons. A value of *P* < 0.05 was used as the criterion for statistical significance.

## Ethics Statement

All animal experimental protocols were approved by the Nanjing University Animal Care Committee and followed the Guide for the Care and Use of Laboratory Animals published by the Chinese National Institutes of Health. The research protocols were conducted in accordance with the animal behavioral guidelines, using approved protocols from the institutional animal care committee (#2014-SR-079). Serum sample collection in this project was approved by the Ethics Committee of Nanjing Drum Tower Hospital, in accordance with the Declaration of Helsinki.

## Author Contributions

LZ and YF performed experiments, analyzed the data, and wrote the initial manuscript. HW and YG performed experiments. WZ, MG, and NZ collected and maintained clinical samples and data. ZW supervised the study and revised the manuscript.

### Conflict of Interest Statement

The authors declare that the research was conducted in the absence of any commercial or financial relationships that could be construed as a potential conflict of interest.

## Supplementary Material

Supplementary Table 1 The miRNA expression profiles in M1 and M2 macrophage-like THP-1 cells infected by SFTSV.
